# Development and in-vitro validation of an intraoral wearable biofeedback system for bruxism management 

**DOI:** 10.3389/fbioe.2025.1572970

**Published:** 2025-10-15

**Authors:** Khalid A. Al-Hamad, Ashwaq Asiri, Ali M. Al-Qahtani, Saud Alotaibi, Abdullah Almalki

**Affiliations:** ^1^ Department of Maxillofacial and Diagnostic Sciences, College of Dentistry, Majmaah University, Majmaah, Saudi Arabia; ^2^ Department of Restorative Dental Sciences, College of Dentistry, Majmaah University, Majmaah, Saudi Arabia; ^3^ Department of Diagnostic Sciences and Oral Biology and Periodontology, King Khalid University, Abha, Saudi Arabia; ^4^ Department of Preventive Dental Sciences, College of Dentistry, Majmaah University, Majmaah, Saudi Arabia

**Keywords:** bite nightguard, oral appliances, bruxism detection, dental health, occlusal splint, occlusion, biosensor technology, biofeedback

## Abstract

**Introduction:**

Bruxism remains a diagnostic challenge, with no consistently reliable clinical approaches available to document the condition with satisfactory accuracy. This study aimed to incorporate a biosensor device into a conventional bite-night guard to detect bruxism in an *in vitro* setting.

**Methods:**

A sandwich-layering process was used to integrate stress and vibration sensors into an acrylic occlusal stabilization splint. The system included a microcontroller, control unit, and data acquisition module. Occlusal force signals were processed using artificial intelligence-based algorithms. A total of 200 repeated trials were conducted to evaluate system performance. Accuracy, sensitivity, and specificity were calculated as validation metrics.

**Results:**

The biosensor prototype demonstrated reliable performance across a force range of 274–700 N. Quantitative evaluation of the neural network yielded an accuracy of 91%, sensitivity of 88%, and specificity of 90% in distinguishing occlusal force thresholds.

**Conclusion:**

The findings confirm the feasibility of integrating biosensors within an intraoral appliance for bruxism detection *in vitro*. Future research should explore long-term durability testing in moist environments and conduct *in vivo* trials to validate clinical performance.

## 1 Introduction

Bruxism is characterized by involuntary movements of the masticatory muscles that induce teeth grinding and clenching, which can result in various dental pathologies (Lantada et al., 2012). It can be classified into two types: sleep bruxism and awake bruxism. The first type occurs during sleep and entails the grinding and clenching of teeth, either rhythmic or nonrhythmic. Awake bruxism manifests during wakefulness and involves recurrent tooth contact and jaw clenching (Balanta-Melo et al., 2019; Chemelo et al., 2020; Zieliński, et al., 2024). Stress is commonly linked to awake bruxism, with an incidence of 22.1%–31% in adults (Nota et al., 2019). The incidence of sleep bruxism is estimated to be 13%, and its prevalence varies widely from 8.6% to 31% (Khoury et al., 2016). Several factors, including gastroesophageal reflux, sleep apnea, family history, anxiety, depression, alcohol consumption, and smoking, may mediate the etiopathogenesis of bruxism (Balanta-Melo et al., 2019; Gizler et al., 2024; Häggman-Henrikson et al., 2024; Zieliński et al., 2024). Despite not being a life-threatening condition, bruxism can considerably affect an individual’s quality of life by causing stress fractures in dental restorations, tooth deterioration, and facial discomfort (Labunet et al., 2022). Currently, no systemic treatments or specific dental procedures, either prosthetic or orthodontic, exist for the effective prevention or treatment of bruxism (Bussadori et al., 2020). This is likely because diagnosing and determining the prognosis of bruxism is challenging (Minakuchi et al., 2022; Minervini et al., 2022).

Typically, diagnosis is based on self-reports and clinical indicators such as tooth wear (Yap and Chua, 2016; Zieliński et al., 2024; Teen et al., 2025). Although no reliable clinical approaches are available to precisely document bruxism with satisfactory diagnostic accuracy (Chemelo et al., 2020; Vieira et al., 2023; Häggman-Henrikson et al., 2024), clinical management includes pharmacological therapies such as muscle relaxants, nonsteroidal anti-inflammatory drugs (NSAIDs), antidepressants, opioids, anticonvulsives, and injection of a local anesthetic agent or botulinum toxin (BOTOX^®^) at trigger points. Non-pharmacological therapies include occlusal splints (OSs), dry needling, physiotherapy and rehabilitation, ultrasound therapy, transcutaneous electrical nerve stimulation, physical self-regulation techniques, acupuncture, stretching exercises, mesotherapy, massage therapy, low-level laser therapy, and behavioral treatment (Teen et al., 2025).

Clinically, monitoring changes in bruxism frequency enables healthcare professionals to adjust treatment approaches and ensure that patients receive appropriate care. Consequently, there has been increasing interest in biofeedback, a method of behavioral modification that uses electronic monitoring for providing a response whenever bruxism is identified (Yamaguchi et al., 2012; Vieira et al., 2023). Developing an intraoral biosensor that seamlessly integrates into a biting guard and includes a vibration module is an alternative to traditional oral bite splints. This approach addresses diagnostic challenges associated with conventional methods and highlights the need for alternative diagnostic and treatment options when traditional techniques prove insufficient. In addition, the integration of other sensors within traditional oral bite splints provides superior benefits and helps patients and physicians track bruxism in real time (Prasad et al., 2023). Triboelectric nanogenerators (TENGs) have gained attention as a promising technology for self-powered health monitoring due to their ability to convert mechanical energy from human motion into electrical signals. Recent advances in flexible electronics and nanomaterials have further enhanced their potential for wearable and integrated applications. With the growing demand for autonomous healthcare devices, TENGs offer a sustainable solution to power constraints while enabling continuous, non-invasive data acquisition. The integration of artificial neural networks (ANNs) further propels this field, unlocking intelligent material design and adaptive signal interpretation for precision medicine (Pan et al., 2025). Therefore, we aimed to develop an intraoral biosensor device that can detect bruxism within a traditional bite guard focusing on biofeedback mechanism.

## 2 Materials and methods

### 2.1 Device design and components

**FIGURE 1:**
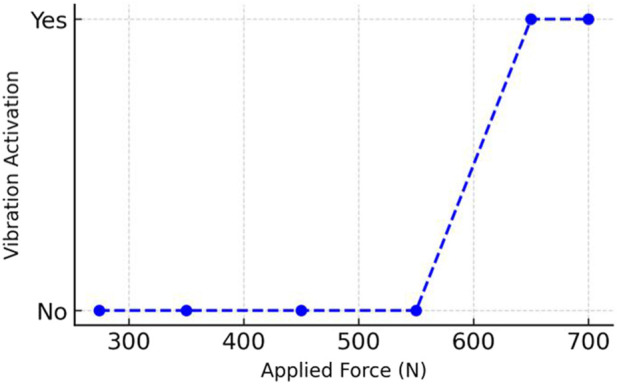
Workflow of the intraoral biosensor system *in vitro*, showing rest mode, pressure sensing, digital acquisition, threshold analysis, and signal transmission via Bluetooth.

**FIGURE 2:**
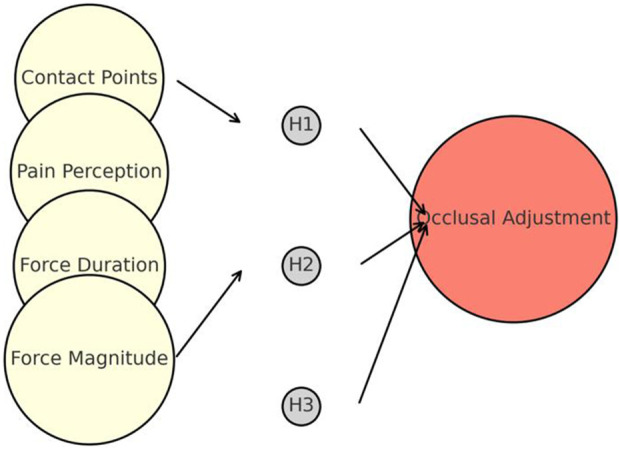
Process map of prototype assessment and integration of the intraoral biosensor device, including AI-based analysis and simulated evaluation of occlusal stress.

The materials used in the development of the biosensor device included a soft vacuum thermoplastic sheet, microcontroller, vibration sensor, and force sensor. This design prioritized low power consumption and triggered data transmission at specific thresholds (Figure 1). Figures 1, 2 depict the system for monitoring bruxism-related behaviors, comprising a pressure sensor, microcontroller transmitter module, and separate vibration sensor.

### 2.2 Fabrication and experimental setup

In this *in vitro* study, we evaluated the device using a dental cast and manual pressure to simulate bruxism. The theoretical outcome of the experiment was the ability of the occlusal splint (OS) to detect pressure and vibration sensor stimulations. We created an OS with a 2-mm breadth on both the buccal and lingual surfaces to ensure secure friction-based attachment. Next, we coated the buccal, occlusal, and lingual surfaces of the entire upper arch with a uniform layer of clear light-curing resin (3M, Z350 XT, St. Paul, MN, United States), approximately 1 mm in thickness. The excess resin was removed from the model using a laboratory knife and cutting along a designated completion line. The stress-sensitive chip and control chip FSR 402 (approximately 0.4 mm each; Interlink Electronics, Inc., Camarillo, CA, United States) were validated using an Arduino Mega2560 system (Arduino S.r.l, Monza, Monza E Brianza, Italy) and correctly positioned at specified positions on the OS. We evenly applied a another coating of transparent resin (approximately 1 mm thick) to coat both the chips and the original resin layer. Subsequently, a light-curing device (EliparTM LED Curing Light; power: 1,200 mW/cm^2^; duration: 40 s; 3M; St. Paul, MN, United States) was used to cure the chips. Final modifications to the occlusal contacts in the optimal contact regions were made, and the device was subjected to surface polishing. In total, five prototype splints were fabricated and tested. Each prototype underwent 200 repeated trials under controlled conditions simulating occlusal loading. Data are presented as mean ± standard deviation (SD). Statistical analysis included descriptive statistics as well as performance metrics (accuracy, sensitivity, and specificity) derived from classification outputs of the machine learning model.

Accurate modification of the occlusal surface is crucial when using a tooth guard to identify clenching. Optimal occlusal sites must meet precise criteria, including the equal distribution of occlusal forces across all posterior and canine teeth during maximum biting. These criteria involve simultaneous bilateral contacts, balanced force sharing, and the absence of premature contacts, which ensure splint stability and minimize localized stress. During normal biting, the posterior teeth in the mandible come into contact with the device, ensuring uniform force distribution, albeit with a somewhat greater pressure than at the anterior teeth. Under no circumstances do the posterior teeth come into contact during forward or lateral motion (Candirli et al., 2016). Uniform contact of the anterior teeth of the mandible with the device is necessary during jaw protrusion. During lateral movement, the device comes into contact with the lower canine teeth exclusively. The signal-sampling module consisted of a sequence of delicate stress-sensitive chips and a control chip, FSR 402 (approximately 0.4 mm thick; Interlink Electronics, Inc., Camarillo, CA, United States). This sensor can provide proportional voltage modulations in response to variation in dental pressure and was calibrated to the measuring device to ensure that the sensor reading is accurate on the digital push–pull force dynamometer.

### 2.3 Data acquisition and processing

The primary function of the main control unit was to receive occlusal force data from a stress-measuring device using a digital push–pull force dynamometer (Changsha Langshuo Technology Co., Ltd., Guangdong, China); this stress-measuring device was programmed for an average maximum bite force of 400–600 N based on a previous study (Calderon Pdos et al., 2006). A measurement test was conducted on the pressure force applied in the range of 274–700 N. The main control unit was responsible for processing, storing, and packaging the collected data. This configuration utilized a low-power microcontroller unit (NRF52832; Nordic Semiconductor, Oslo, Norway) as the primary controller (Naresh and Lee, 2021). The system oversaw the collection of several stress signals, converted the analog signals into digital format using a multichannel analog-to-digital converter module (SAADC, Nordic Semiconductor, Oslo, Norway), and sent the processed data for full analysis. A button battery (Panasonic Semiconductor, Panasonic CR2032, Celebes, Indonesia) provided the power supply for the control unit with a continuous operational lifespan. The central processing unit module was engineered to automatically shut down when the battery no longer supplied the required operational voltage. The primary mode of transmission was a wire, which processed occlusal force data from a microcontroller unit to a server terminal, which transformed the exceeding or average frequent occlusal force signals into vibratory stimulus signals; if the occlusal force signals exceeded 600 N, the vibratory stimulus signals would prompt the vibration. The objective of the biofeedback system was to induce a learned response for mitigating bruxism without necessitating the use of a feedback system.

**FIGURE 3:**
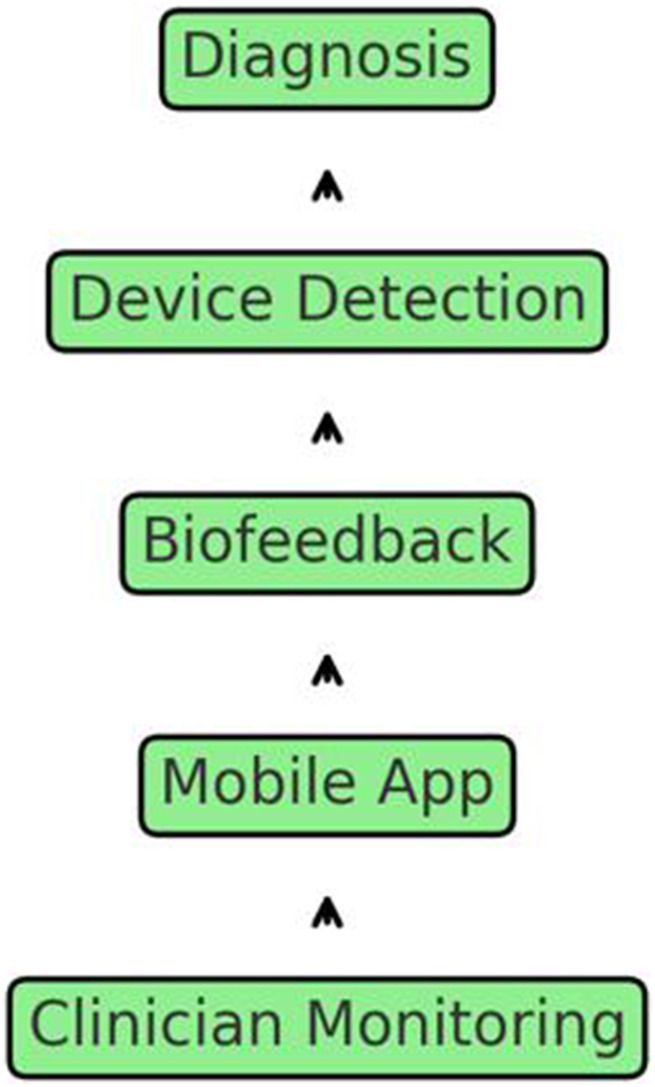
Comprehensive schematic of the bruxism management system, illustrating diagnosis, device detection, biofeedback, mobile app integration, and remote clinician monitoring.

In addition, the display provided information on the bite duration, force dispersion, and contact, allowing real-time self-care tracking. Furthermore, it disseminated and interpreted data, thereby enabling remote and continuous monitoring in clinical practice. Figure 3 illustrates the procedure of the proposed biofeedback system.

This process facilitated instantaneous updates of occlusal data. The server terminal module used a conventional server (Hewlett-Packard Development Company, HP Z840, Beijing, China) with a graphics processing unit coprocessor (NVIDIA, TiTan XP, Santa Clara, CA, United States) to train the machine learning algorithms.

### 2.4 Statistical analysis and machine learning techniques

Using a machine learning technique, the server primarily analyzed the biting force data and presented curves derived from the pressure data collected by the sensors. An occlusal splint must be introduced into a semi-adjustable articulator for providing real-time and flexible monitoring of biting forces. This splint stimulates the patient’s occlusion and aligns it accurately with the dental structures. Therefore, the entire system must exhibit attributes such as small dimensions, low energy consumption, and reliable performance. The primary control unit, serving as the central element, formed the essential core of the entire detection assembly, thereby determining the capacity of the system to reliably and accurately detect and transmit data.

**FIGURE 4:**
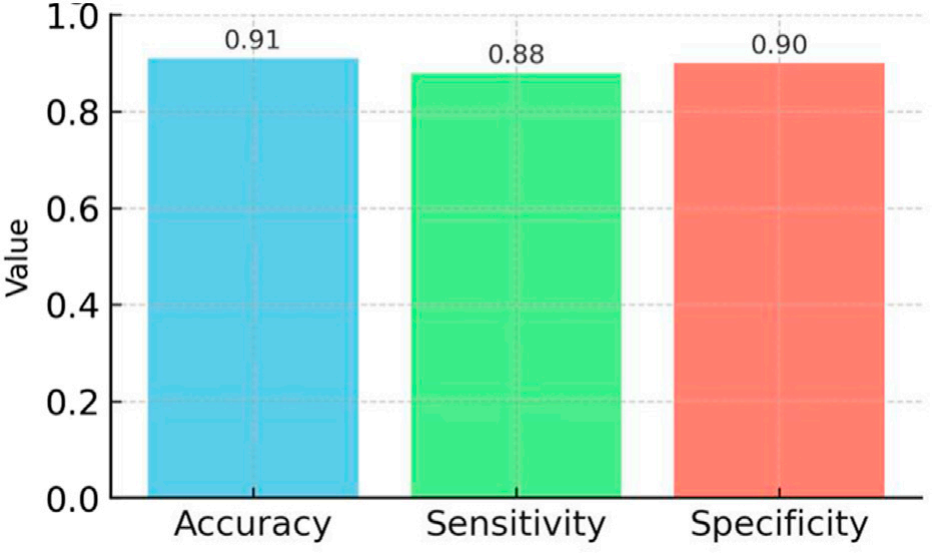
Artificial neural network model architecture for occlusal force analysis, showing input parameters (force contact points, pain perception, duration, magnitude), hidden layers, and predicted level of occlusal adjustment.

The hyperparameters were tuned using a programming language Python 2.7.18 (Python software foundation, Beaverton, OR, United States). The statistical methodology focused on five essential components: (1) the biting force duration, represented by the mean value (AVGxi); (2) the bite-induced force, which exhibits the maximum (MAXxi) and minimum (MINxi) magnitudes; (3) teeth contact on occlusal surface, which exerts a force on the stress sensor; (4) change in the voltage of the sensor output; and (5) the analog-to-digital converter using the open-source Arduino software (IDE) 1.8.19 (Arduino S.r.l, Monza, Monza E Brianza, Italy) receives the aforementioned modifications to facilitate data conversion. We ensured the precise measurement of the start time of the force to avoid erroneous positive outcomes. Feedback analysis was used to evaluate the biofeedback process. The microcontroller unit is triggered if the digital signal input from the analog-to-digital converter exceeds a predetermined threshold, indicating an abnormal occlusion event. The multichannel analog-to-digital conversion-processing port-discovery module is then activated by incorporating the operating procedures outlined in Figure 4.

**FIGURE 5:**
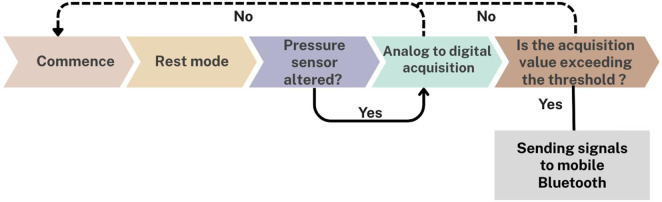
Relationship between applied occlusal force (N) and vibration sensor activation during *in vitro* testing of the intraoral prototype.

This study used wavelet transformation to denoise the data signals. This procedure involves converting a signal influenced by both the noise and stress characteristics into a wavelet domain. Next, we separated the wavelet modification of the signal from the noise. We eliminated the noise transformation coefficients and subjected the remaining coefficients to an inverse transformation to generate a denoised signal. We converted the data signal representing the occlusion stress into the wavelet domain using the wavelet transform procedure. Wavelet transformation-based denoising techniques often employ a translation-invariant wavelet denoising approach to denoise the objectives. For example, if the original signal X(t) is recorded between 0 and t ≤ n and the signal Sh(t) = X (t + h) is created by time-domain translation with a positive integer, then T (x; (Sh){h\in H_n}) = Ave{h\in H_n} (T (Sh)) for n-cycle translation can be used for denoising; where (H_n = {h: 0 ≤ t ≤ n}), Ave denotes the average value, and (T (Sh)) represents the denoising process for signal S using the Donho threshold method. Using the Powerdrill tool (Rocosky Technology Pte., Ltd., Singapore), occlusal force data analysis enabled the extraction of crucial factors relevant to occlusion modification over time using the Python linear regression artificial intelligence (AI) method. The parameters included the mean occlusion force magnitude, mean occlusal force duration, mean contact area generated by the occlusal forces, and contact points resulting from the occlusal force. Multiple experimental sessions were conducted throughout the project to determine the grade of the operator. A horseshoe-shaped articulating paper with a 40-micron thickness was used to measure the contact area and number of occlusal contact sites. Figure 5 illustrates the proposed implementation of a one-dimensional deep convolutional neural network for intelligent analysis and diagnostic support.

This technique employed a residual neural network architecture to improve the testing accuracy of the network. In this context, the terms “pwconv1” and “pwconv2” denote one-dimensional pointwise convolutions, whereas “dwconv” represents a one-dimensional depth wise convolution. The abbreviation for batch normalization is “BN,” whereas “Relu6″ is a nonlinear activation function. As illustrated in Figure 4, the neural network generates an output that accurately represents the degree of occlusion correction. Each level corresponded to a precise degree of increase or decrease in the height of the occlusion adjustment, usually approximately 0.5 mm. Next, we trained the neural model using the initial experimental values and training data. Composed of >50,000 training data points, the neural model learns to determine a suitable level of occlusion adjustment based on unknown inputs. This can offer valuable guidance to dentists regarding occlusion adjustments.

This study suggests several methods for determining the final occlusion adjustment. Before training the neural network algorithm, a technique was required to calibrate the input and corresponding occlusion adjustments. This initial step allowed the algorithm model to learn the network parameters from the calibrated dataset and analyze the real data based on the acquired model parameters. The training process of the neural network for testing purposes involved the use of a momentum gradient descent method with a training coefficient of 0.9 and batch size of 128. Moreover, transfer learning was used to pretrain the network with external data, which improved the initialization weights before training. We established 80 sets of sample calibration training data in a specific implementation to improve the accuracy of the algorithm and set aside an additional 20 sets of sample inference data for testing. The dataset used to train the neural network comprised data collected from different occlusions in the same semi-adjustable articulator. By analyzing the real-time stress data per unit time from the stimulator, a specialist can identify the occlusion behavior and severity levels, which will facilitate diagnosis and treatment.

### 2.5 Biofeedback mechanism

During the initial evaluation, we established the initial parameters and thresholds for the occlusion detection algorithm by examining bite conditions in various occlusion scenarios. In our proposed scenario, we equipped patients with a monitor and gathered real-time occlusion stress data using a predetermined threshold. These data served as the foundation for generating feedback responses.

**FIGURE 6:**
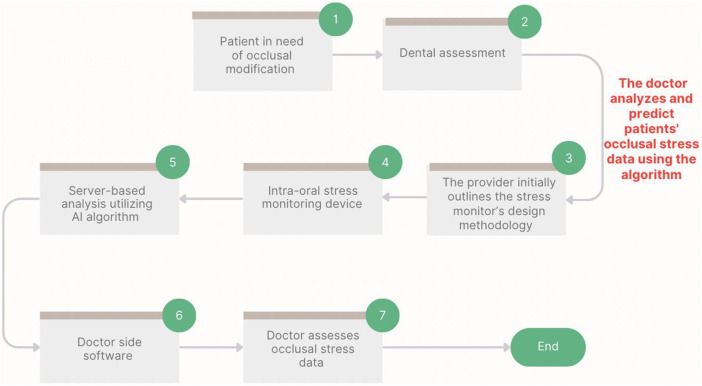
Performance metrics (accuracy, sensitivity, specificity) of the neural network model trained on occlusal force data, based on 200 repeated *in vitro* trials, demonstrating reliable detection of bruxism-related events.

Bluetooth transmission technology facilitates the transfer of occlusion data to mobile devices. Here, the mobile application employes an integrated algorithm to monitor real-time changes in occlusion. Consequently, patients receive notifications based on the findings of the algorithm, which prompt them to assess their current occlusion stress condition and record their existing pain levels in the mobile application. The application records the patients’ input and sends the accumulated data to the server for detailed analysis. The AI algorithm processes the occlusion analysis results of the patient on the server. Doctors can access the occlusion stress data through a dedicated mobile application. These findings enable dynamic real-time modification of parameter threshold values during the biofeedback treatment process, even from a remote location. This real-time adjustment enables precise numerical biofeedback monitoring and logging (Figure 6).

## 3 Results

We successfully created a prototype system for an biosensor, which incorporated a set of strain-sensitive film sensors and a microprocessor. The dimensions of the prototype were approximately 21 × 17.5 mm. Upon integrating the circuit board into the OS, we fine-tuned and omitted various test pads, resulting in a scaled-down size of 6 × 6 mm. We then inserted the microprocessor-printed circuit board into a testing board enable with external devices through analog-to-digital converter pins, thereby verifying the functionality of the pressure signal-acquisition process. We independently acquired, transmitted, and received the stress signal data. Data from the resistive thin-film pressure of the force sensor, which served dual purposes as both input and output signals for subsequent statistical analysis modules. The experiment revealed that the input voltages showing the occlusal forces were very low, and large changes in the occlusal forces were recorded by the piezoresistive film stress sensor. This information shows the extent of the adverse impact of bruxism episodes. Our denoising algorithm eliminated unwanted noise data from the pressure signal collection module. Quantitative evaluation of the neural network yielded an accuracy of 91%, sensitivity of 88%, and specificity of 90% in distinguishing occlusal force thresholds. These metrics confirm the feasibility of the prototype *in vitro*, but we refrain from extrapolating to clinical use without further validation.

Despite the significant noise produced by the pressure signal collection module, it remained effective. The occlusion-adjustment level of the neural network, which is crucial for the detection and potential management of bruxism through the biofeedback system, was valid for successful data transfer, as the trial was conducted 200 times (Table 1). This is significant, because it serves as the primary source of feedback outputs. As demonstrated through several cycles, both the input signals and feedback indicators collected for analysis at any given time can be used to detect bruxism in clinical settings. Table 2 summarizes the main comparative features of the intraoral biosensor prototype and other devices for bruxism management, including validation metrics (accuracy, sensitivity, and specificity). Table 3 expands this comparison by incorporating additional intraoral devices reported in the literature, underscoring the unique integration of biofeedback, AI, and denoising algorithms in the present prototype.

**TABLE 1:** Mean occlusal force values (N) recorded across posterior and canine sites under maximum biting conditions, with corresponding vibration sensor activation outcomes. Data represent mean ± SD from 200 repeated *in vitro* trials using five intraoral biosensor prototypes.

Force (N)	Vibration sensor stimulated? (yes/no)
274	No
450	No
500	No
550	No
600	No
650	Yes
700	Yes

**TABLE 2:** Comparative features of the intraoral biosensor prototype versus existing intraoral devices for bruxism management, showing the presence or absence of bruxism detection, biofeedback, and AI integration. Validation metrics (accuracy, sensitivity, specificity; Mean ± SD) from 200 *in vitro* trials are reported for the prototype.

Device	Bruxism detection	Biofeedback	Integration with AI	Validation (Mean ± SD)
Conventional Occlusal Splint	No	No	No	-
Quad Helix	Limited	No	No	-
s-Guard	Yes	Yes	No	-
Current Prototype	Yes	Yes (sensor + vibration)	Yes	Accuracy 91% ± 2%; Sensitivity 88% ± 3%; Specificity 90% ± 2%

**TABLE 3:** Detailed comparison of the present intraoral biosensor prototype with various existing intraoral devices for bruxism management, highlighting unique advantages such as real-time occlusal force measurement, AI-driven analysis, and denoising algorithms. The table contrasts design purpose, feedback mechanism, and data acquisition capabilities based on previously reported devices in the literature.

Type of intraoral device	Description
Occlusal splint (Kaya et al., 2022)	Although traditional occlusal splints provide passive protection against bruxism, our prototype further ensures active monitoring of occlusal forces with precise data collection and real-time feedback, which makes it potentially more informative for targeted treatment
Quad Helix (de Moura, 2023)	Designed for children, the Quad Helix focuses on altering occlusal patterns but lacks the advanced data acquisition that our biosensor provides, which could enable a more dynamic response to real-time bruxism events
Biofeedback devices (Pfeiffer, 2021)	Similar in concept (both devices aim to reduce bruxism behavior through feedback); however, the strain-sensitive sensors of our device enable more detailed data collection on occlusal force variation, which enhances accuracy in monitoring bruxism severity
s-Guard (Câmara-Souza, 2020)	Similar to the s-Guard, our biosensor collects data and supports Internet of Things connectivity. However, the denoising algorithm of our device improves data fidelity, which is critical for accurate analysis and feedback
Vibratory feedback devices (Nakazato et al., 2021)	Although vibratory feedback devices aim to stop bruxism without disrupting sleep, our prototype provides direct occlusal force measurement, which enhances the precision of feedback and potentially reduces false signals during rest periods
Electrical stimulation devices (Aqueveque, 2013)	These use direct stimulation, whereas our device records occlusal data for analysis and intervention. The strain-sensitive sensors of our biosensor allows continuous, noninvasive monitoring rather than immediate physical correction
Diagnostic and treatment devices (Tanaka Lozano, 2015)	Our prototype similarly monitors bruxism but stands out by integrating a neural network to refine feedback outputs, thereby providing a more specific and responsive biofeedback mechanism compared conventional diagnostic and therapeutic tools

## 4 Discussion

Over the years, extensive discussion has been conducted on utilizing biofeedback for tooth grinding (Ohara et al., 2022). Our study demonstrates an intraoral biosensor embedded in a night guard for *in vitro* detection of bruxism. Key limitations include restriction to bench testing only, lack of long-term durability testing in moist conditions, and absence of *in vivo* clinical validation. We therefore recommend future studies to test the prototype under realistic oral environments, perform larger *in vivo* trials, and evaluate long-term stability and patient comfort. The discussion of clinical applicability has been reduced to avoid overstatement, and results are interpreted conservatively.

We developed the entire sensor-system integration approach *ab initio*, considering both hardware and firmware designs. Placing a full-dentition occlusion detector on the mandibular or maxillary dentition allows real-time dynamic monitoring of biting forces. The detection system was based on a primary control module. Therefore, it is small, reliable, cost effective, and easy to implement. In this study, we created a central component that governs the functionality of the entire detection system, which is the primary control module responsible for ensuring accurate and reliable data identification and transmission. The digital sensor of the device employed 1 MB of flash memory and 256 KB of RAM, and its onboard memory was 2 MB of QSPI flash (Majithia et al., 2015). One of the primary challenges is the effective integration of a sensor chip into a bite guard to ensure its consistent operation and prevent it from moving. This is because incorporating stress sensor devices and embedding them in the mouth can lead to various issues (Gholampour et al., 2019). In this study, we used traditional techniques and polymethyl methacrylate material for manually fabricating an OS. However, the development of digital technologies has recommended additive (printing) or subtractive (milling) methods for fabricating nightguards using polymethyl methacrylate-based resins as the best options (Huettig et al., 2017; Gholampour et al., 2019; Sumonsiri et al., 2019). With this new technology, which includes newly developed intraoral scanners, it is possible to create exact three-dimensional copies of dental curves without creating extra holes. These impression systems provide sufficient clinical precision and higher patient satisfaction than traditional impression methods, allowing for simple interactions with computer-aided design and computer-aided manufacturing printers and a completely digital workflow (Dedem and Türp, 2016; Berntsen et al., 2018). High temperature and stress conditions can compromise the performance of stress-sensing chips (Dedem and Türp, 2016; Ma et al., 2017; Sfondrini et al., 2018; Hao et al., 2023).

In the current study, we hypothesized that, if possible, an intraoral biosensor device could be specifically designed to detect bruxism within a traditional bite guard. The primary challenge we addressed was the successful integration of conventional materials, which required high-temperature and high-stress manufacturing, with chips that cannot endure these conditions. Neither traditional procedures nor digital technologies have yet found a good way to incorporate chips into the resins used in open-source software (Gu et al., 2015; Nakamura et al., 2019). This study employed light curing to enhance the OS, and future studies could explore new avenues in digital technology manufacturing and similar resin selection. Large *in vivo* studies and those implementing appropriate monitoring intervals to assess specific attributes such as clinical reliability, validity, sensitivity, and specificity are also warranted.

We used an AI algorithm to adjust the occlusal force, which is one of the key features highlighted in this study. Machine learning models use the average occlusal force, duration, contact area, contact point, and biofeedback at the pressure level to determine the degree of occlusion modification. Therefore, the experiment included both the inputs and outputs in the results. Machine learning models require extensive training using abundant datasets for generating more accurate results. In this study, we presented a novel concept and developed a crude prototype to integrate machine learning with an AI algorithm, aiming to integrate them into future clinical practice.

However, our study has a limitation. We did not perform any *in vivo* studies. Consequently, we displayed some system demos using a single array of sensors and the associated management mechanisms. Furthermore, the sampling frequency of the input signal was sufficiently high for accommodating lower frequencies of bruxism. We collected all data for our study from real sensors and their corresponding control devices. In addition, the possibility of unequal occlusal forces on both sides due to variations in age, sex, and habitual movement cannot be ruled out. Furthermore, research on this concept is challenging and warrants thorough experimental verification. Our results add to the field of biofeedback knowledge by providing early *in vitro* results that may yield advantageous insights for future research on future bruxism management strategies. Notably, recent advancements in triboelectric nanogenerators (TENGs) have demonstrated their promising potential as self-powered sensors in medical health monitoring applications, enabling real-time and non-invasive physiological signal detection. As highlighted in the review by (Pan et al., 2025), TENG-based devices can convert biomechanical energy into electrical signals with high sensitivity, offering innovative solutions for continuous health monitoring and personalized treatment. Integrating such technologies into biofeedback systems could significantly enhance the development of therapeutic devices targeting conditions like bruxism (Ohara et al., 2022).

In conclusion, in this study, we proposed a comprehensive approach for detecting and potentially improving bruxism management using an occlusion stress sensor system. Our research focused on integrating and embedding stress sensors across a full set of teeth using a sandwich method. We tested and validated this approach by incorporating occlusal force data processing using a machine learning algorithm. The experimental results of force detection for bruxism detection and monitoring using realistic parameter measurements obtained from the sensor. Future steps include the development of a clinically optimized device with acceptable intraoral dimensions, followed by double-blind clinical trials to confirm long-term reliability, validity and patient safety.

## Data Availability

The original contributions presented in the study are included in the article/supplementary material, further inquiries can be directed to the corresponding author.
